# Scale Insects, edition 2, a tool for the identification of potential pest scales at U.S.A. ports-of-entry (Hemiptera, Sternorrhyncha, Coccoidea)

**DOI:** 10.3897/zookeys.431.7474

**Published:** 2014-08-06

**Authors:** Douglass R. Miller, Alessandra Rung, Grishma Parikh

**Affiliations:** 1 Systematic Entomology Laboratory, Agricultural Research Service, U.S. Department of Agriculture, Beltsville, MD 20705, USA; 2 Plant Pest Diagnostics Branch, California Department of Food and Agriculture, Sacramento, CA 95832, USA; 3 California Agricultural Support Services, Sacramento, CA 95811, USA

**Keywords:** Agricultural pest, Coccoidea, Interactive key, Identification tool, Lucid, Scale insects

## Abstract

We provide a general overview of features and technical specifications of an online, interactive tool for the identification of scale insects of concern to the U.S.A. ports-of-entry. Full lists of terminal taxa included in the keys (of which there are four), a list of features used in them, and a discussion of the structure of the tool are provided. We also briefly discuss the advantages of interactive keys for the identification of potential scale insect pests. The interactive key is freely accessible on http://idtools.org/id/scales/index.php

## Introduction

Scale insects include notorious invasive species. They are small, often live in concealed portions of their hosts, and are transported in commodities that are common in international trade (Miller et al. 2002). Scales cause billions of dollars in damage to U.S. crops and in control costs annually (Kosztarab 1977, 1990; Kondo et al. 2008) and nearly all damaging scale pests are species that were inadvertently introduced (Miller et al. 2002; Miller and Miller 2003).

Only a few specialists in the world can identify scale insects based on morphology. Of these, many are retired or approaching retirement, putting scale insect systematics in danger of being stifled by inadequate manpower (Kondo et al. 2008; Hardy 2013). Moreover, because scale insect pests may arrive from all over the world (Miller et al. 2014), regional expertise and scale insect diagnostic tools with a regional focus are inadequate for border patrol (Hardy 2013). Considering the common presence of scales in interceptions (Evans and Dooley 2013), we have produced a user-friendly, online tool that can be used at U.S.A. ports-of-entry to help with the identification of potentially invasive species.

Although many dichotomous keys have been developed for scales (Hardy 2013), they are regional in scope and do not have the flexibility of interactive keys. For instance, in traditional dichotomous keys, features appear in a fixed order, whereas in Lucid-based and other interactive keys, there are many paths to a correct identification. This is advantageous because some features are difficult to interpret, difficult to see on the specimen in hand, or are absent in a damaged specimen. Another benefit of interactive keys over dichotomous keys is that the user can express uncertainty by selecting more than one state per character, and view/modify the character states already selected (Walter and Winterton 2007; Cerretti et al. 2012; Dallwitz et al. 2013).

An earlier version of Scale Insects comprising 148 species was published on CDROM and uploaded to the USDA web site in 2007 (Miller et al. 2007). It was mentioned as useful by Hardy (2013) and has been part of training programs for state and federal identifier workshops, being often used by U.S. port personnel for making identifications (Greggory Evans, APHIS identifier, personal communication, 2014). The new edition includes 46 additional species, and updated information on the distribution of previously included species. Furthermore, the key to families was updated with new findings in the higher classification of scale insects (e.g., Hodgson 2012). The new tool also differs from the old one in how the glossary (pages containing pest-specific information) and the fact sheets are generated: in the old tool, fact sheets and the glossary are static html pages, whereas in the new tool they are dynamically generated and thus can be more easily updated.

As far as we know, one other Lucid key, with a regional scope, is available online to identify scales (Hardy and Kondo 2009). The general structure of Scale Insects, edition 2 emulates a number of Lucid-based tools for pest identification created and/or funded by the USDA Animal and Plant Health Inspection Service (APHIS), Identification Technology Program (ITP), and which can be found at http://idtools.org/id/. Our tool differs from all of them by being extensively linked to a comprehensive database on scale insects, ScaleNet (Ben-Dov et al. 2012). ScaleNet is unusual in that it is not only a specimen database *per se*, but a complex model of the scale insect literature (*c.* 25,000 references) (Hardy 2013). The links to ScaleNet give the user instant access to updated taxonomy, distribution and host records, and literature citations.

Despite the usefulness of Scale Insects, edition 2, it must be emphasized that it contains only a small subset of the world scale fauna. According to ScaleNet there are 7,792 species of scale insects worldwide. Therefore, a species that is not represented in the tool may key out as one that is (a false positive identification). For this reason, identifications should always be checked against information on the fact sheets and compared against authoritatively identified reference specimens for further assessment.

The following groups are represented in Scale Insects, edition 2 (Miller et al. 2014): Scale Families, encompassing the families most often encountered at the U.S.A. ports-of-entry; Soft Scales; Mealybugs and Mealybug-like families; and Other Scales, encompassing pests in various families with the exclusion of the Diaspididae. The tool makes extensive use of hypertext to link to images, glossaries, and other support material, and has four main components: Lucid-based interactive keys, fact sheets, glossary of morphological terms, and image gallery.

## Format of the paper

This paper was written following the outlines for data papers provided by Penev et al. (2009, 2012) and the format was inspired on Cerretti et al. (2012) and Chrétiennot-Dinet et al. (2014).

## Project description

There are four keys in Scale Insects, edition 2: Scale families, Mealybugs and Mealybug-like families, Soft Scales, and Other Scales (details for each key provided below). Terminal taxa in these keys are potential pests to U.S.A. agriculture, from all over the world, and were selected from a list of interceptions by APHIS-Plant Protection and Quarantine (PPQ) between 1995 and 2013. This list can be obtained from APHIS upon request, but has not been published. Below we describe the taxonomic coverage of each key, and provide lists of the features used to discriminate taxa in them. Features in the keys are from the adult female, are standard in scale insect taxonomy (Kondo et al. 2008) and, for the most part, can only be seen on a slide-mounted specimen.

## Scale Families key

### General features

The key matrix is based on 66 morphological features of the adult female and includes 33 extant families as terminal taxa.

### List of the terminal taxa included in the current version

Aclerdidae; Asterolecaniidae; Beesoniidae; Callipappidae; Carayonemidae; Cerococcidae; Coccidae; Coelostomidiidae; Conchaspididae; Dactylopiidae; Diaspididae; Eriococcidae, Halimococcidae; Kermesidae; Kerriidae; Kuwaniidae; Lecanodiaspididae; Marchalinidae; Margarodidae; Matsucoccidae; Micrococcidae; Monophlebidae; Ortheziidae; Phenacoleachiidae; Phoenicococcidae; Pityococcidae; Polliniinae; Pseudococcidae; Putoidae; Rhizoecidae; Steingeliidae; Stictococcidae; Xylococcidae.

### Features used in the key

The morphological features to separate among scale families are listed in Table 1. They were largely taken from the works mentioned below, from isolated descriptions, and analysis of specimens: Aclerdidae (McConnell 1954); Asterolecaniidae (Stumpf and Lambdin 2006); Cerococcidae (Lambdin and Kosztarab 1977); Coccidae (Hodgson 1994); Conchaspididae (Mamet 1954); Dactylopiidae (De Lotto 1974); Diaspididae (Danzig 1993; Takagi 1960); Eriococcidae (Kozár et al. 2013); Kermesidae (Bullington and Kosztarab 1985); Kerriidae (Chamberlin 1923); Lecanodiaspididae (Borchsenius 1960; Howell and Kosztarab 1972); Margarodidae (Jakubski 1965); Monophlebidae (Morrison 1928); Ortheziidae (Kozár 2004); Pseudococcidae (Williams and Granara de Willink 1992); Putoidae (McKenzie 1967); Rhizoecidae (Kozár and Konczné Benedicty 2007); Stictococcidae (Richard 1971, 1976).

**Table 1.** Features used in the Key to Scale Insect families of Scale Insects, edition 2. Features are listed according to the main region of the body where they occur. “General features” are present in at least two different main body regions. Abbreviations: QP, quinquelocular pores.

Location in body	Features
General features	Cerarii (presence); marginal setae (presence); size of abdominal spiracles with respect to thoracic spiracles; tubular ducts (presence; shape of tubular duct invagination), invaginated tubular duct in QP clusters (presence); 8-shaped pores (presence), predominant pore type; ornate setae over dorsum (presence).
Head	Number of labial segments. Number of antennal segments, campaniform sensilla on second antennal segment (presence and number), antennal bar (presence and shape), length of basal antennal segment with respect to others, basal antennal segment sclerotization (presence), reticulate pattern of antenna (presence), antennal articulatory process on first segment (presence), shape of apical antennal segment, enlarged seta on apex of antenna (presence).
Thorax	Size of first pair of thoracic spiracles with respect to second pair; bar or sclerotized area on spiracle (presence); pores in atrium of thoracic spiracles (presence and number); thoracic spiracles close to anal opening (presence); ocellar spot (presence); row of pores in spiracular furrow (whether defined or not); spiracular setae (presence); metasternal sclerotization (presence). Legs. Whether present, developed or reduced; length of front legs with respect to mid and hind legs; trochanter pores on each surface (presence and number), distribution of trochanter pores; fusion between trochanter and femur (presence); coxal structure, whether divided longitudinally or not; fusion between tibia and tarsus (presence); number of setae on hind tibia; clubbed setae on distal end of tibia (presence); number of tarsal segments, shape of tarsus, clubbed tarsal digitules (presence, arrangement), campaniform sensilla on tarsus (presence); claw (presence), claw digitules (presence and shape), claw denticles (presence, shape and number), basal claw denticle (presence); translucent pores on hind legs (presence).
Abdomen	Number of abdominal spiracles; relative size of first six abdominal spiracles with respect to last two; pores in abdominal spiracles (presence); anal opening position; anal tube sclerotization (degree, position); number of anal ring setae; anal ring pores (presence and location); anal fig(s) (presence, number, position); anal cleft (presence); arch fig (presence); lateral sclerotized bar near anal ring (presence); anal lobes (shape) and presence of setae on projecting anal lobes; anal opening position on body; cicatrices (presence); cribriform figs (presence); circulus (presence); dorsomedial spine anterior of anal ring (presence); marginal crenulations (presence); ostioles (presence), pygidium (presence); vulvar orientation.

## Soft Scales

### General features

The key matrix is based on 41 morphological features and includes 48 species in 21 genera of Coccidae.

### List of the terminal taxa included in the current version

*Ceroplastes ceriferus* (Fabricius, 1798); *Ceroplastes cirripediformis* Comstock, 1881; *Ceroplastes floridensis* Comstock, 1881; *Ceroplastes japonicus* Green, 1921; *Ceroplastes rubens* Maskell, 1893; *Ceroplastes rusci* (Linnaeus, 1758); *Ceroplastes sinensis* Del Guercio, 1900; *Ceroplastes stellifer* (Westwood, 1871); *Coccus capparidis* (Green, 1904); *Ceroplastes hesperidum* Linnaeus, 1758; *Ceroplastes longulus* (Douglas, 1887); *Ceroplastes moestus* De Lotto, 1959; *Ceroplastes pseudohesperidum* (Cockerell, 1895); *Ceroplastes viridis* (Green, 1889); *Drepanococcus chiton* (Green, 1909); *Eucalymnatus tessellatus* (Signoret, 1873); *Kilifia acuminata* (Signoret, 1873); *Kilifia americana* Ben-Dov, 1979; *Kilifia deltoides* De Lotto, 1965; *Marsipococcus proteae* (Brain, 1920); *Megapulvinaria maxima* (Green, 1904); *Milviscutulus mangiferae* (Green, 1889); *Parasaissetia nigra* (Nietner, 1861); *Parthenolecanium corni* (Bouché, 1844); *Phalacrococcus howertoni* Hodges & Hodgson, 2010; *Philephedra broadwayi* (Cockerell, 1896); *Philephedra lutea* (Cockerell, 1893); *Philephedra tuberculosa* Nakahara & Gill, 1985; *Prococcus acutissimus* (Green, 1896); *Protopulvinaria longivalvata* Green, 1909; *Protopulvinaria pyriformis* (Cockerell, 1894); *Pseudokermes vitreus* (Cockerell, 1894); *Pulvinaria floccifera* (Westwood, 1870); *Pulvinaria hydrangeae* Steinweden, 1946; *Pulvinaria ixorae* Green, 1909; *Pulvinaria polygonata* Cockerell, 1905; *Pulvinaria psidii* Maskell, 1893; *Pulvinaria urbicola* Cockerell, 1893; *Pulvinariella mesembryanthemi* (Vallot, 1829); *Saissetia coffeae* (Walker, 1852); *Saissetia miranda* (Cockerell & Parrott, 1899); *Saissetia neglecta* De Lotto, 1969; *Saissetia oleae* Olivier, 1791; *Taiwansaissetia formicarii* (Green, 1896); *Tillancoccus tillandsiae* Ben-Dov, 1989; *Tillancoccus mexicanus* Ben-Dov, 1989; *Udinia catori* (Green, 1915); *Udinia farquharsoni* (Newstead, 1922).

### Features used in the key

The morphological features to separate among soft scale pests are listed in Table 2. They were largely extracted from the comprehensive works of De Lotto (1965), Gill (1988), Gill et al. (1977), Gimpel et al. (1974), Hamon and Williams (1984), Hodgson (1994), and Williams and Watson (1990), from isolated descriptions, and analysis of specimens.

**Table 2.** Features used in the Key to Soft Scales of Scale Insects, edition 2. Features are listed according to the main region of the body where they occur. “General features” are present in at least two different main body regions. Abbreviations: MP, multilocular pores.

Location in body	Features
General features	Body shape; dermal reticulation in mature females (presence); dorsal setae (presence, shape, thickness); filamentous ducts on body margin (presence); marginal setae thickness, shape at apex; tubular ducts (presence overall), distribution on venter, presence on dorsum; submarginal tubercles (presence and distribution).
Head	Number of long setae between antennae. Number of antennal segments.
Thorax	Stigmatic setae (whether differentiated or not, length, number); number of rows of stigmatic setae, whether rows contiguous between spiracles or not; MP anterior of anterior spiracle (presence, size with respect to other MPs); number of marginal setae between anterior spiracular furrows. Legs. Whether present and developed; cavity on mid and hind coxae (presence); claw denticle (presence); tibio-tarsal sclerosis (presence); tibio-tarsal spur on hind leg (presence and development); relative sizes of claw digitules.
Abdomen	Anal fig (shape, position, number of apical, subapical, discal and subdiscal setae); anal fig protuberance (presence); fringe setae (total number); preopercular pores (presence, distribution); number of elongate prevulvar setae; MP distribution, number of loculi in MP near vulva.

## Mealybugs and Mealybug-like families

### General features

The key matrix is based on 44 morphological features and covers 99 species in 27 genera and three families (96 species in 24 genera in Pseudococcidae, two species in one genus of Putoidae, and one species and one genus of Rhizoecidae).

### List of the terminal taxa included in the current version

**Pseudococcidae** (Mealybugs): *Antonina graminis* (Maskell, 1897); *Antonina nakaharai* Williams & Miller, 2002; *Balanococcus diminutus* (Leonardi, 1918); *Brevennia rehi* (Lindinger, 1943); *Chaetococcus bambusae* (Maskell, 1893); *Crisicoccus azaleae* (Tinsley, 1898); *Delottococcus aberiae* (De Lotto, 1961); *Delottococcus confusus* (De Lotto, 1977); *Dysmicoccus boninsis* (Kuwana, 1909); *Dysmicoccus brevipes* (Cockerell, 1893); *Dysmicoccus grassii* (Leonardi, 1913); *Dysmicoccus lepelleyi* (Betrem, 1937); *Dysmicoccus mackenziei* Beardsley, 1965; *Dysmicoccus neobrevipes* Beardsley, 1959; *Dysmicoccus orchidum* Williams, 2004; *Dysmicoccus* sp. nr. *texensis*; *Dysmicoccus sylvarum* Williams & Granara de Willink, 1992; *Dysmicoccus wistariae* (Green, 1923); *Exallomochlus camur* Williams, 2004; *Exallomochlus hispidus* (Morrison, 1921); *Exallomochlus philippinensis* Williams, 2004; *Ferrisia dasylirii* (Cockerell, 1896); *Ferrisia malvastra* (McDaniel, 1962); *Ferrisia terani* Williams & Granara de Willink, 1992; *Ferrisia virgata* (Cockerell, 1893); *Formicococcus njalensis* (Laing, 1929); *Formicococcus polysperes* Williams, 2004; *Formicococcus robustus* (Ezzat & McConnell, 1956); *Hordeolicoccus heterotrichus* Williams, 2004; *Hordeolicoccus nephelii* (Takahashi, 1939); *Hypogeococcus pungens* Granara de Willink, 1981; *Laminicoccus pandani* (Cockerell, 1895); *Maconellicoccus hirsutus* (Green, 1908); *Maconellicoccus multipori* (Takahashi, 1951); *Nipaecoccus annonae* Williams & Granara de Willink, 1992; *Nipaecoccus jonmartini* Williams & Granara de Willink, 1992; *Nipaecoccus nipae* (Maskell, 1893); *Nipaecoccus viridis* (Newstead, 1894); *Palmicultor browni* (Williams, 1960); *Palmicultor palmarum* (Ehrhorn, 1916); *Paracoccus burnerae* (Brain, 1915); *Paracoccus ferrisi* Ezzat & McConnell, 1956; *Paracoccus herreni* Williams & Granara de Willink, 1992; *Paracoccus interceptus* Lit, 1997; *Paracoccus lycopersici* Ezzat & McConnell, 1956; *Paracoccus marginatus* Williams & Granara de Willink, 1992; *Paracoccus mexicanus* Ezzat & McConnell, 1956; *Paracoccus solani* Ezzat & McConnell, 1956; *Paraputo guatemalensis* (Ferris, 1953); *Paraputo odontomachi* (Takahashi, 1951); *Paraputo olivaceus* (Cockerell, 1896); *Phenacoccus defectus* Ferris, 1950; *Phenacoccus franseriae* Ferris, 1921; *Phenacoccus gossypii* Townsend & Cockerell, 1898; *Phenacoccus hakeae* Williams, 1985; *Phenacoccus helianthi* (Cockerell, 1893); *Phenacoccus madeirensis* Green, 1923; *Phenacoccus parvus* Morrison, 1924; *Phenacoccus solani* Ferris, 1918; *Phenacoccus solenopsis* Tinsley, 1898; *Phenacoccus stelli* (Brain, 1915); *Planococcus citri* (Risso, 1813); *Planococcus ficus* (Signoret, 1875); *Planococcus halli* Ezzat & McConnell, 1956; *Planococcus kraunhiae* (Kuwana, 1902); *Planococcus lilacinus* (Cockerell, 1905); *Planococcus litchi* Cox, 1989; *Planococcus minor* (Maskell, 1897); *Pseudococcus aurantiacus* Williams, 2004; *Planococcus baliteus* Lit, 1994; *Planococcus calceolariae* (Maskell, 1879); *Planococcus comstocki* (Kuwana, 1902); *Planococcus cryptus* Hempel, 1918; *Planococcus dendrobiorum* Williams, 1985; *Planococcus elisae* Borchsenius, 1947; *Planococcus jackbeardsleyi* Gimpel & Miller, 1996; *Planococcus landoi* (Balachowsky, 1959); *Planococcus longispinus* (Targioni Tozzetti, 1867); *Planococcus lycopodii* Beardsley, 1959; *Planococcus maritimus* (Ehrhorn, 1900); *Planococcus microcirculus* McKenzie, 1960; *Planococcus nakaharai* Gimpel & Miller, 1996; *Planococcus odermatti* Miller & Williams, 1997; *Planococcus philippinicus* Williams, 2004; *Planococcus pithecellobii* Gimpel & Miller, 1996; *Planococcus solenedyos* Gimpel & Miller, 1996; *Planococcus viburni* (Signoret, 1875); *Rastrococcus iceryoides* (Green, 1908); *Rastrococcus invadens* Williams, 1986; *Rastrococcus spinosus* (Robinson, 1918); *Saccharicoccus sacchari* (Cockerell, 1895); *Spilococcus mamillariae* (Bouché, 1844); *Vryburgia amaryllidis* (Bouché, 1837); *Vryburgia distincta* (De Lotto, 1964); *Vryburgia succulentarum* Williams, 1985; *Vryburgia viator* (De Lotto, 1961). **Putoidae** (Giant mealybugs): *Puto barberi* (Cockerell, 1895); *Puto mexicanus* (Cockerell, 1893). **Rhizoecidae** (Ground mealybugs): *Rhizoecus amorphophalli* Betrem, 1940.

### Features used in the key

When the user first opens the Mealybugs and Mealybug-like Families key, he/she will be prompted to choose among the following families in the features window (in order to use this key, the user must know the family classification of the specimen): Pseudococcidae, Rhizoecidae or Putoidae. After the appropriate family has been selected, the features that differentiate among the species included in that family will open. While most features separate among species of Pseudococcidae, one character (presence/absence of tubular ducts in cerarii) is used to separate between the two species of Putoidae. This character was only coded for putoids. Since Rhizoecidae is represented by only one species (above), choosing this family will automatically result in species identification.

Morphological features used to separate among species of Pseudococcidae are listed in Table 3 and were largely taken from the comprehensive works of Ferris (1950), McKenzie (1967), Miller and McKenzie (1973), Miller (1975), Cox (1987, 1989), Williams and Watson (1988), Kosztarab (1996), Williams and Miller (2002), Miller and Giliomee (2011), Kaydan and Gullan (2012), Williams (2004), from isolated descriptions, and the analysis of specimens. Features to separate Putoidae species were taken from Williams and Granara de Willink (1992).

**Table 3.** Features used to separate Pseudococcidae species in Scale Insects, edition. Features are listed according to the main region of the body where they occur. “General features” are present in at least two different main body regions. Abbreviations: DP, discoidal pores, MP, multilocular pores, ORTD, oral-rim tubular ducts, OCTD, oral-collar tubular ducts, TP, trilocular pores.

Location in body	Features
General features	Cerarii (presence, number on each side of body); ORTD (presence overall, presence on venter, number on dorsum); MP on dorsum (presence); OCTD on dorsum (presence); TP (presence and position); *Ferrisia*-like rim around tubular ducts (presence), position of DP in rim of *Ferrisia*-like tubular ducts, position of setae in rim of *Ferrisia*-like tubular ducts, sclerotization around *Ferrisia*-like tubular ducts (named A and B in key); QP (presence, position)
Head	Head: ORTD (presence on frontal dorsal region, presence on dorsomarginal region between cerarii 15-16); cerarii on head and/or prothorax (presence); DP near eye (presence); number of OCTD between antennae. Number of antennal segments
Thorax	Thorax: ORTD (number laterad of mid coxa, presence of cluster between front coxa and body margin); spiracular pores (presence); DP on derm surrounding hind coxa (presence); MP (presence on dorsal mid-thorax). Legs (presence); translucent pores on hind legs (presence and position); denticle on claw (presence)
Abdomen	Abdomen: anal bar (presence); auxiliary filamentous setae in 2^nd^ cerarius (presence); conical setae in abdominal cerarii (presence); circulus (presence, shape); presence of dorsomedial cerarii; shape of cerarian setae; number of conical setae in anal lobe cerarii; setae on dorsum of segment VIII longer than on segments VII and VI (presence); shape and length of dorsal setae; MP (presence on ventrolateral abdominal portion, on abdominal segments I-VIII); dorsal ORTD (presence near lateral margin of most abdominal segments, number on abdomen)

## Other scales

### General features

The key matrix is based on 41 morphological features and covers 47 species (entities) in 26 genera and 11 families.

### List of the terminal taxa included in the current version

Family names, in bold, are not terminal taxa, but were added below to help situate the different species in the higher classification.

**Aclerdidae** (Flat grass scales): *Aclerda takahashii* Kuwana, 1930; *Aclerda sacchari* Teague, 1925. **Asterolecaniidae** (Pit scales): *Asterolecanium epidendri* (Bouché, 1844); *Bambusaspis bambusae* (Boisduval, 1869); *Bambusaspis miliaris* (Boisduval, 1869); *Palmaspis inlabefacta* (Russell, 1941); *Palmaspis phoenicis* (Ramachandra Rao, 1922); *Planchonia stentae* (Brain, 1920); *Russellaspis pustulans* (Cockerell, 1892); *Sclerosococcus ferrisi* McKenzie, 1958; *Sclerosococcus tillandsiae* Lambdin, 1980. **Conchaspididae** (False armored scales): *Conchaspis angraeci* Cockerell, 1893; *Conchaspis capensis* (Linnaeus, 1763); *Conchaspis orchidarum* Mamet, 1954. **Dactylopiidae** (Cochineal scales): *Dactylopius coccus* Costa, 1829; *Dactylopius confusus* (Cockerell, 1893); *Dactylopius opuntiae* (Cockerell, 1896); *Dactylopius tomentosus* (Lamarck, 1791). **Eriococcidae** (Felt scales): *Asiacornococcus kaki* (Kuwana, 1931); *Acanthococcus araucariae* (Maskell, 1879); *Acanthococcus coccineus* (Cockerell, 1894); *Acanthococcus dubius* (Cockerell, 1896); *Ovaticoccus agavium* (Douglas, 1888). **Kerriidae** (Lac scales): *Kerria lacca* (Kerr, 1782); *Paratachardina pseudolobata* (Kondo & Gullan, 2007). **Lecanodiaspididae** (False pit scales): *Lecanodiaspis dendrobii* (Douglas, 1892); *Lecanodiaspis prosopidis* (Maskell, 1895); *Psoraleococcus multipori* (Morrison, 1921). **Matsucoccidae** (Pine bast scales): *Matsucoccus feytaudi* Ducasse, 1941; *Matsucoccus josephi* Bodenheimer & Harpaz, 1955; *Matsucoccus matsumurae* (Kuwana, 1905). **Monophlebidae** (Giant scales): *Crypticerya genistae* (Hempel, 1912); *Crypticerya rosae* (Riley & Howard, 1890); *Drosicha* sp.; *Icerya aegyptiaca* (Douglas, 1890); *Icerya pulchra* (Leonardi, 1907); *Icerya purchasi* Maskell, 1879; *Icerya samaraia* (Morrison, 1927); *Icerya seychellarum* (Westwood, 1855). **Ortheziidae** (Ensign scales): *Insignorthezia insignis* (Browne, 1887); *Insignorthezia pseudinsignis* (Morrison, 1952); *Newsteadia floccosa* (De Geer, 1778); *Ortheziola vejdovskyi* Šulc, 1895; *Praelongorthezia praelonga* (Douglas, 1891). **Stictococcidae** (Stictococcids): *Stictococcus formicarius* Newstead, 1910; *Stictococcus intermedius* Newstead, 1917; *Stictococcus sjostedti* Cockerell, 1903.

### Features used in the key

As with the Key to Mealybugs and Mealybug-like families, the first feature of this key is family, of which there are 11. Therefore, to use this key the user needs to determine the family first, using the Key to families. Morphological features used to separate among species of the families listed below are presented in Table 4, and were largely taken from the literature (cited below after each family):

**Aclerdidae:** McConnell (1954). **Asterolecaniidae:** Stumpf and Lambdin (2006). **Conchaspididae:** Mamet (1954). **Dactylopiidae:** De Lotto (1974). **Eriococcidae:** Kozár et al. (2013). **Kerriidae:** Chamberlin (1923)**. Lecanodiaspididae:** Borchsenius (1960), Howell and Kosztarab (1972). **Matsucoccidae:** Foldi (2005). **Monophlebidae:** Morrison (1928). **Ortheziidae:** Kozár (2004). **Stictococcidae:** Richard (1971, 1976). Additional features were taken from isolated descriptions, and analysis of specimens.

**Table 4.** Features used in the Key Other Families of Scale Insects, edition 2. Features are listed according to the main region of the body where they occur. “General features” are present in at least two different main body regions. Abbreviations: DP, discoidal pores, QP, quinquelocular pores.

Family	Features
Aclerdidae	Dorsal or ventral microducts on marginal areas of head, thorax, and abdomen (dorsal or ventral position).
Asterolecaniidae	dorsal 8-shaped pores (presence and position, sizes, excluding lateral ones); marginal 8-shaped pores (presence); dorsal 8-shaped tubular ducts (presence); submarginal DPs (presence); MP distribution on ventromedial areas; submarginal QP row between antennae (presence, whether complete); pear shaped anal lobe sclerotization (presence); dorsal tubes (presence); number of setae on each side of anal ring; sclerotization of apex of abdomen (degree).
Conchaspididae	Number of MPs on abdominal segment 3; tubular ducts (presence).
Dactylopiidae	Size of enlarged setae in longitudinal lines; tubular ducts in QP cluster (presence); anal ring sclerotization (presence).
Eriococcidae	QP on dorsum (presence); arrangement of enlarged setae (presence); bifurcation of microtubular duct orifice (presence); number of antennal segments; anal ring pores (presence)
Kerriidae	Shape of body
Lecanodiaspididae	Two long setae anterior of vulva (presence); number of cribriform figs on each side of the body.
Matsucoccidae	Enlarged setae on 5th antennal segment (presence); number of rows of cicatrices
Monophlebidae	Open center pores (presence); cicatrices (number, size); number abdominal spiracles; ovisac band or marsupium band (presence); marsupium (presence)
Ortheziidae	Sclerotization on head (presence, width); number of antennal segments; dorsomedial wax figs medially (presence and reach to marginal figs); bands of spines within ovisac band (presence); fusion between tibia and tarsus (presence)
Stictococcidae	Enlarged marginal seta fringing (presence and number of projections); dorsal submarginal seta fringing (presence); shape of dorsal submarginal seta

## Technical specifications

**Web location:** http://idtools.org/id/scales/index.php

**Platform:** a website

**Web Server:** CentOS

**Programming language:** PHP 5 and MySQL

**Application version:** 2.0

**Data base:** MySQL

**Data:** 2.0

**Language:** English

**License for use of the key:** Attribution-Non-commercial

**Use of the primary data:** available upon request.

The keys were directly built in Lucid builder (various versions up to 2007), then updated on Builder 3.5 (available at http://lucidcentral.org, Queensland, Australia) for the current version. A list of the Lucid3 key files (key data files and key program files) can be found in Penev et al. (2009).

Keys were deployed online using the Lucid Key Server (available at http://lucidcentral.org). The On-line Player is an alternative to the Lucid3 Application Player for interactive keys created using the Lucid3 Builder. Playing keys using the Lucid On-line Player does not require Java installation. This method may be advantageous to government workers because Java applications are often partially or totally blocked in government computers due to security concerns. Furthermore, some keys deployed using the Lucid3 Player may temporarily stop working or work defectively after a Java update, or may not work properly until an update has been implemented. However, it must be emphasized that the not all features of the Lucid3 Player are available in the On-line Player. For instance, the function to “prune redundant features” has been implemented in the former but not in the latter. Moreover, while the help icon (?) of the Lucid 3 Player is linked to a help file online, the help icon of the Lucid Online Player is linked to the Lucid webpage on (http://www.lucidcentral.org/en-us/software/lucid3.aspx); from there the user has to find the help file that has been written for the Lucid3 player. Despite those caveats, we believe that the On-line Player is a work in progress, and that in the long run the advantages of using it will outweigh the disadvantages of the Java player. A thorough discussion of the differences between the two programs for playing Lucid keys is warranted.

Information in fact sheets and glossary is managed using the Fact Sheet Manager (FSM), an interface for a database that stores all the data present in the dynamic pages of the website (fact sheets and glossary of terms). Both FSM and the database have been created and are maintained by the Identification Technology Program (ITP), USDA APHIS PPQ. When the user chooses an entity (by selecting a link of a particular species), a single fact sheet page that is coded with queries to the database populates the appropriate fact sheet content. This contrasts with the static HTML fact sheets in the previous version of Scale Insects (Miller et al. 2007), and allows for quick updates and corrections that go online almost immediately.

## Tool details

**Figure 1.**
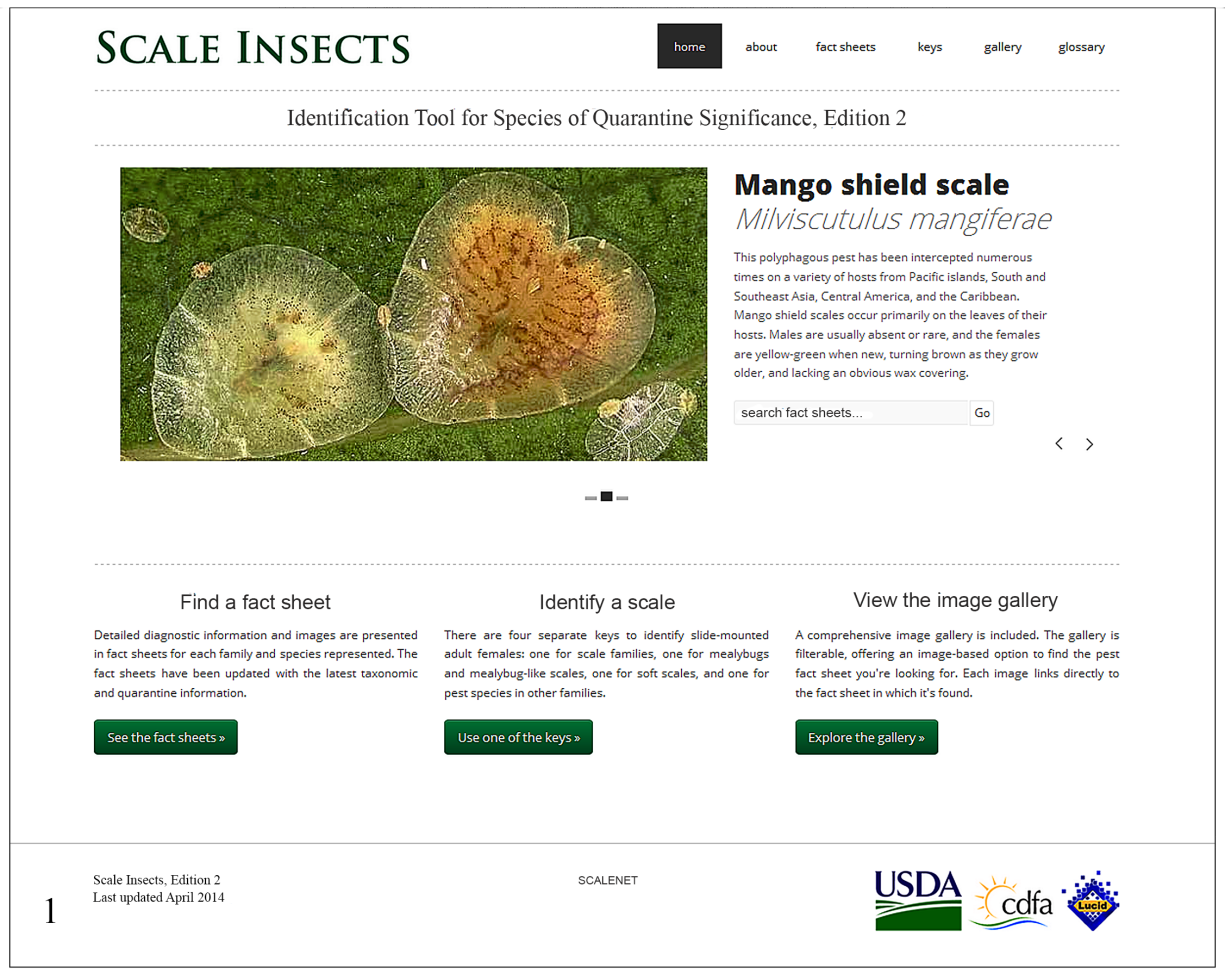
Screen snapshot of the home page of Scale Insects, edition 2 (viewed from Firefox 27.0.1 on April 10, 2014).

In the portal Scale Insects, edition 2, the user has the option to find a fact sheet, use one of the keys, view the image gallery or consult the glossary of morphological terms (Fig. 1).

**Figure 2.**
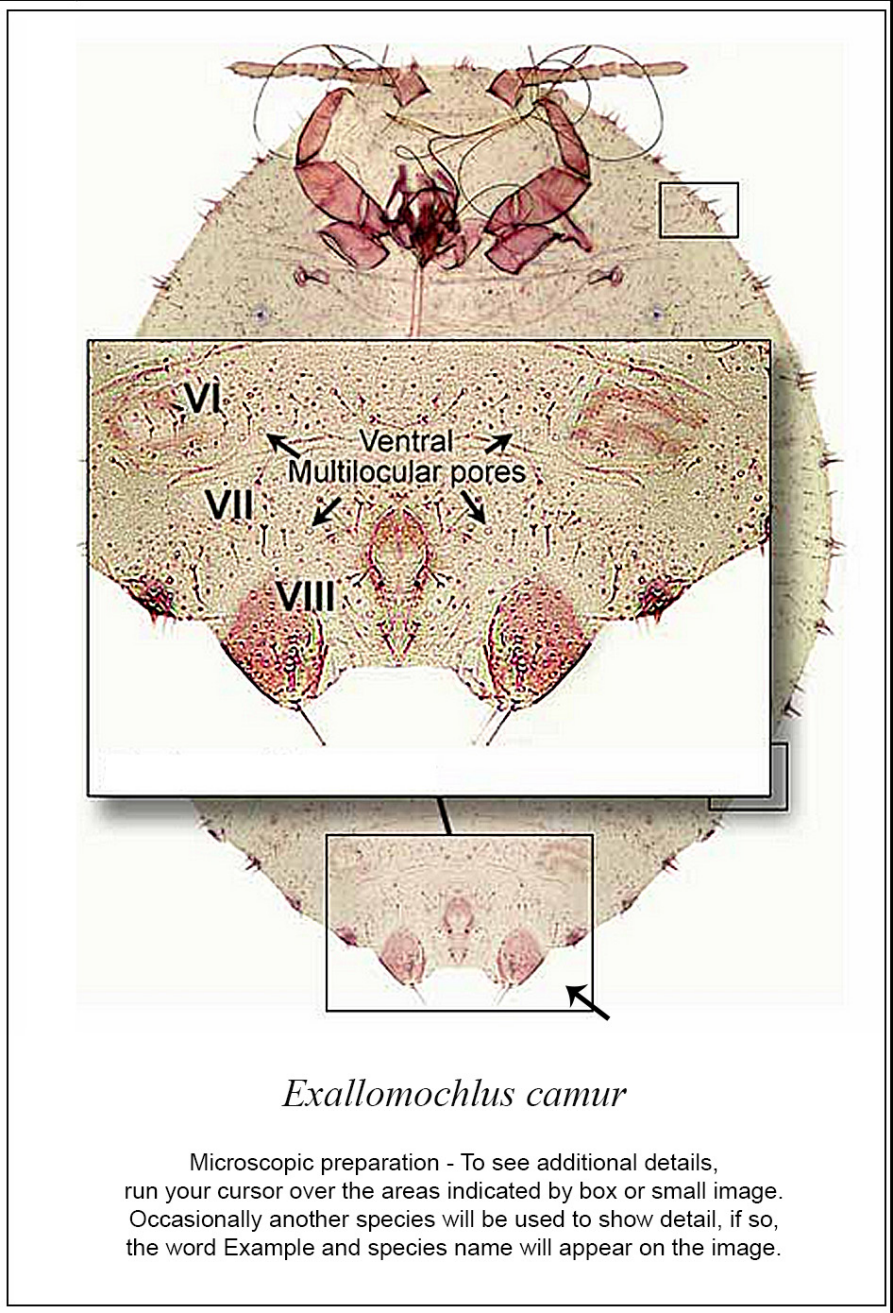
Screen snapshots of the diagnostic page of *Exallomochlus camur* Williams in the Mealybugs and Mealybug-like families of Scale Insects, edition 2 (viewed from Firefox 27.0.1 on April 10, 2014). Diagnostic features are marked by rectangles; rolling the mouse over each rectangle will bring up an enlargement of the feature.

Searching for a family in the “search fact sheets” box will list a fact sheet for that family and for the corresponding species in one of the keys, if present. For instance, a search for Aclerdidae will find three fact sheets, one for Aclerdidae in the Families key and two for species of *Aclerda* Signoret in the Other Scales key. Fact sheets in keys to species contain the following information: a link to a Catalog on ScaleNet, Common name, Field characters (diagnostic description), Validation characters (diagnostic description of the adult female), Comparison (with similar species), U.S.A. quarantine notes, and Important References (a link to ScaleNet). In fact sheets, most morphological terms are hyperlinked to the glossary, and two or three figures are present, which can be enlarged by clicking on them: a line drawing with structures labeled, a habitus picture and a picture of the slide mounted, whole body of a mature female. In the enlargement of the whole body, diagnostic features are marked with squares. When the user scrolls the mouse over a square, an enlargement of the corresponding feature will pop up in a window (Fig. 2).

The image gallery offers a quick way to perform identifications by matching the specimen in hand with a drawing or a photograph. Images can be filtered in two ways: by including or excluding images of the habitus, whole body picture or drawing; and by selecting a package (each package corresponds to a Lucid key and associated fact sheets and images).

**Figure 3.**
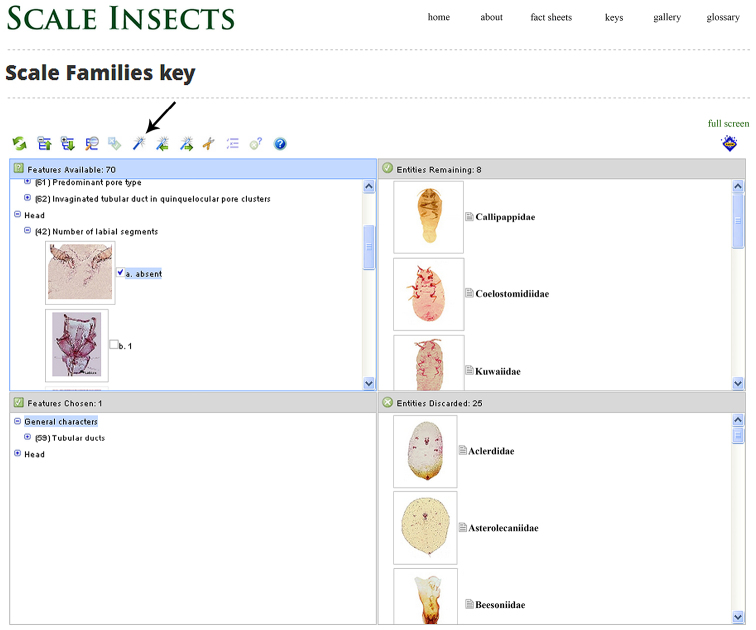
Screen snapshot of the Lucid Key to families, Scale Insects, edition 2 (viewed from Firefox 27.0.1 on April 10, 2014). Features can be selected in any order or they can be selected by the program by clicking on the “best character” icon (indicated with black arrow). Entities with the character states selected remain in the “entities remaining” window (upper right window) whereas those that do not match the states selected are sent to the “entities discarded” (lower right window).

From the Lucid On-line Player the Lucid key looks very similar to its Lucid3 Player counterpart (Fig. 3). The terminal taxa are represented as Entities on the right windows, whereas the features are represented as Features on the left windows. When a feature state is selected, the entities that do not have that feature will be moved into the “Entities Discarded” window (lower right), and the character state selected will be moved to the lower left window. All entities and feature states are richly illustrated with photographs and/or drawings. Clicking on the image thumbnail of a state brings up a larger image. Clicking on the taxon thumbnail brings up a larger window with thumbnails of a line drawing with important structures labeled, a whole body picture, and a habitus picture (when available). Clicking on the name of the taxon brings up a link to the fact sheet for that taxon.

## Conclusions

Protecting the borders of large countries such as the United States from invasive scales often requires a very broad knowledge of the taxonomy the group, and consultation of books and papers that are scattered all over the place. Scale Insects, edition 2 may facilitate the job of target users, which include USDA APHIS pest survey specialists, identifiers at ports of entry, state and county identifiers, students, and scientists, in three ways. First, it condenses, in one online resource, a wide array of information on target species from various zoogeographical regions. Second, it maintains current taxonomic information through links to ScaleNet, a relational database that is updated regularly. Third, additional species of concern can be easily added to the Lucid keys and fact sheets. We believe that our tool will facilitate insect pest identifications and we hope that it will inspire taxonomists in other groups to build similar tools.
